# COVID-19 and Pregnancy: Vertical Transmission and Inflammation Impact on Newborns

**DOI:** 10.3390/vaccines9040391

**Published:** 2021-04-15

**Authors:** Mohamed Joma, Claire-Maelle Fovet, Nabila Seddiki, Pierre Gressens, Mireille Laforge

**Affiliations:** 1 Université de Paris, NeuroDiderot, Inserm, 75019 Paris, France; mohamed.joma@st.ul.edu.lb (M.J.); pierre.gressens@inserm.fr (P.G.); 2 INSERM U1184, CEA, IDMIT Department, Immunology of Viral, Auto-Immune, Hematological and Bacterial Diseases (IMVA-HB), Université Paris-Saclay, 92265 Fontenay-aux-Roses, France; claire-maelle.fovet@cea.fr (C.-M.F.); nabila.seddiki@cea.fr (N.S.)

**Keywords:** COVID-19, fetus, newborn, transmission, immunity, inflammation, preterm delivery, central nervous system, neurodevelopmental disorders

## Abstract

The COVID-19 pandemic is ongoing and we are still compiling new findings to decipher and understand SARS-CoV-2 infection during pregnancy. No reports encompass any conclusive confirmation of vertical transmission. Nevertheless, cases of fetal distress and multiple organ failure have been reported, as well as rare cases of fetal demise. While clinicians and scientists continue to seek proof of vertical transmission, they miss the greater point, namely the cause of preterm delivery. In this review, we suggest that the cause might not be due to the viral infection but the fetal exposure to maternal inflammation or cytokine storm that translates into a complication of COVID-19. This statement is extrapolated from previous experience with infections and inflammation which were reported to be fatal by increasing the risk of preterm delivery and causing abnormal neonatal brain development and resulting in neurological disorders like atypical behavioral phenotype or autistic syndrome. Given the potentially fatal consequences on neonate health, we highlight the urgent need for an animal model to study vertical transmission. The preclinical model will allow us to make the link between SARS-COV-2 infection, inflammation and long-term follow-up of child brain development.

## 1. Introduction

In December, 2019, several cases of pneumonia with an unknown cause were detected in Wuhan, China [1]. On 7 January 2020, investigators identified the etiological agent as a novel coronavirus, initially designated as 2019n-CoV, which was later changed to Severe Acute Respiratory Syndrome Coronavirus-2 (SARS-CoV-2) [2]. On February 11, 2020, the World Health Organization (WHO) named the disease resulting from this novel viral infection Coronavirus Disease-19 (COVID-19) [3]. As of March 30, 2021, 127,258,173 individuals had been diagnosed, and 2,785,286 deaths had been recorded, according to an epidemiological study conducted at John Hopkins University [4]. The clinical symptoms of COVID-19 patients have been found to be associated with several comorbidities such as preexisting cardiovascular diseases, diabetes, hypertension, and diseases of the respiratory system [5,6,7]. The most common clinical features include fever, cough, fatigue, and headache [8]. The R_0_, or basic reproduction number, for COVID-19 is 2 to 3.5, in part because approximately 80% of patients who are asymptomatic nevertheless release large amounts of viral particles, which makes it challenging to contain the spread of infection [9]. Certain factors can increase the risk of viral infection, including pregnancy which puts the pregnant woman and her fetus at high risk for infection. In this review, we investigated the maternal and fetal complications, the possibility for vertical transmission, the effects of cytokine storm and inflammation on the fetus, and the potential neurological teratogenic effects of the virus during pregnancy that could cause additional neural complications in the brain development of the fetus. We also described the utility of animal models, especially the nonhuman primate (NHP) models to study vertical transmission during COVID-19 to better understand if the fetus is prone to in utero infection by the SARS-CoV-2 or if its exposure to an inflamed environment through maternal immune activation (MIA) is sufficient to be fatal and could lead to neurodevelopment disorders in the long-term.

## 2. Methodology

Articles were selected from different databases with the following keywords: SARS-CoV-2, COVID-19, viral and vertical transmission, pregnancy, neonatal, cytokine storm, inflammation, neurodevelopment, complications, animal model. No language restriction was placed on the published articles. The interpretation and conclusion we came up with are based on literature analysis together with our expertise in the field of infection and inflammation. The duration was from December 1, 2019 to March 2021 for extracting the literature and screening the articles of potential interest.

## 3. Maternal Manifestations and COVID-19

The physiological system of a pregnant woman adapts to the embryo implantation, and this includes several anatomical and physiological changes. For instance, pregnant women often undergo cardiac changes, including an increase in cardiac output by 20%, an increase in pulmonary vascular resistance, the development of systemic vascular resistance, which could result in hypertension [10], and an increase in hemoglobin demand due to neonatal oxygen needs [10]. Recent studies have shown that SARS-CoV-2 attacks β-chain hemoglobin, detaching iron from porphyrin, decreasing oxygen and carbon dioxide transport, and contributing to oxygen supply failure [11], which translates into severe multiple organ failure in neonates [12,13]. Pregnancy is also accompanied with the elevation of the diaphragm to make room for the fetus, thus decreasing tolerance of hypoxia [14]. Moreover, alveolar damage due to an excessive immune response, which releases protease and reactive oxygen species, results in the desquamation of alveolar cells added to pulmonary edema, which limits gas exchange [15]. The mother’s immune system is locally suppressed at the mother/fetal interface during pregnancy to avoid fetal rejection. Cell-mediated immune response is compromised in order to tolerate the fetus, thus rendering the mother more susceptible to infections [16].

Current knowledge on the effects of SARS-CoV-2 infection in pregnancy comes from case reports and case series studies as well as from population surveillance systems in high-income countries. These studies have focused particularly on pregnant women with symptomatic disease, where deaths have been reported in around 1% of cases [17]. A large data analysis from the PAN-COVID registry (January 1st to July 25th 2020), which includes pregnancies with suspected or confirmed maternal SARS-CoV-2 infection at any stage in pregnancy, and the AAP SONPM National Perinatal COVID-19 registry (April 4th to August 8th 2020), which includes pregnancies with positive maternal testing for SARS-CoV-2 from 14 days before delivery to 3 days after delivery, showed that maternal death was higher than expected based on UK and US population data [18].

Most reports of mothers infected with SARS-CoV-2 indicated that births by cesarean delivery were undertaken to eliminate the risk of neonate infection. The decision of others to undergo cesarean delivery was due to fetal distress [19,20]. Pregnant women presented several morbidity symptoms, but the most common were fever, dry cough, and dyspnea. Blood tests of these women showed lymphocytopenia and elevated C-reactive protein levels (CRP) [21]. However, no reports have been published on neonatal infection through vaginal delivery, and all reported cases of vaginal delivery showed negative Reverse transcription polymerase chain reaction (RT-PCR) results [19,22,23,24,25].

A large amount of reported literature has suggested the possibility of vertical transmission based on RT-PCR analyses on babies born to SARS-CoV-2 positive mothers, but none of these studies gave formal proof of vertical transmission [26,27,28,29,30,31,32,33]. A recent retrospective observational study gathering information from December 1, 2019 to May 15, 2020 and accounting for 688 babies born to 843 SARS-CoV-2 positive mothers was conducted to seek proof of vertical transmission. Vertical transmission was confirmed in a small number of cases, so they suggested that SARS-CoV2 can be vertically transmitted, but the likelihoods very small [34].

## 4. Immunological Aspects of COVID-19 in Babies

The immune system is responsible for the eradication of the virus in the body [35,36,37] directing researchers to develop strategies to boost immune responses, for example, through Interferon Type I (IFN-I) [38]. To support this idea, recent studies conducted by JL Casanova showed that inborn errors of IFN-I immunity accounted for life-threatening COVID-19 pneumonia. In these patients, adaptive autoimmunity impaired innate and intrinsic antiviral immunity by producing neutralizing autoantibodies that impaired IFN-I immunity [39,40]. Knowing that due to the immaturity of their adaptive immune response, neonates receive passive immunity from the mother through the transfer of maternal Immunoglobulin class G (IgG) via the placenta and of Immunoglobulin class A (IgA) via breastfeeding [41,42], such neutralizing autoantibodies against IFN-I could reach the fetus and thus impair virus elimination. Moreover, several other important parameters for immune response development are lacking early in life, and these include (i) neutrophil deficiency in number and function [43], (ii) antigen-presenting cells that have a reduced expression of major histocompatibility complex type II (MHCII), resulting in impaired antigen presentation to T helper cells [44] and reduced cytokine response. Toll Like Receptor (TLR) response in premature infants has also been shown to induce dominant anti-inflammatory innate cytokine Interleukin 10 (IL-10) relative to the term infants’ response, which produces IL-10, Interleukin 6 (IL-6) and Interleukin 23 (IL-23) [45]. 

The first and second trimester are very critical for the fetus during vertical transmission as these account for most complications following viral infections. For instance, the risk for complications from congenital cytomegalovirus (CMV) is higher in the first trimester [46]. Indeed sensorineural hearing loss occurred in 80% of neonates infected with CMV in the first trimester, 8% of those infected in the second trimester, and none in those infected in the third trimester [47]. Likewise, rubella causes most of the severe damages during the first trimester [48]. The primary maternal rubella infection of the mother in the first trimester causes heart problems and sight and hearing problems. However, after 20 weeks of pregnancy, there are usually no complications [49]. It can be noted that congenital viral infections and infections with other viruses, such as the Zika virus show increased rates of infection during the first two trimesters [50].

For these reasons, cohort studies were performed on pregnant women infected with SARS-CoV-2 either in their first or second trimester, to seek the correlation between miscarriage and COVID-19. Cosma S et al. and La Cour Freiesleben N et al. came to the conclusion that there was no correlation between infection in the first trimester and miscarriage referring to their cohort studies [51,52,53]. However Shende P et al. reported the case of an asymptomatic pregnant woman tested positive for SARS-CoV-2 at her 8th week of gestation who ended up negative at 13th week of gestation with fetal demise revealed by ultrasonography. Viral Ribonucleic Acid (RNA) was detected along with Spike 1 (S1) and Spike 2 (S2) viral proteins in the placenta associated with extensive leukocyte infiltration. This was the first study with evidence of persistent placental SARS-CoV-2 infection after maternal cure, suggesting that the virus might reside in the weakest immunocompromised area, namely the placenta, in pregnant women [54].

The first published report of a pregnant woman infected in her second trimester showed a miscarriage during the infection [55]. Before labor, the ultrasound image showed active movement by the fetus and fetal tachycardia. Vaginal swabs were negative to SARS-CoV-2 or bacterial infection, and further investigation for SARS-CoV-2 contamination of the placenta through the vaginal canal was null. Swabs from the fetal mouth, axillae, blood, thymus, liver, lung, umbilical cord blood and amniotic fluid, were negative. However, the placental submembrane and placental cotyledon were positive for SARS-CoV-2, although they were sterile for bacterial or fungal infections. Moreover, placental histology showed inflammatory cell extravasation, including neutrophils and macrophages. The cause of the miscarriage was thus assigned to SARS-CoV-2 because no other reason for fetal demise was found. More recent studies reported positive RNA for SARS-CoV-2 from vaginal swab, and was the first study to detect SARS-CoV2 in breast milk [56]. 

### Antibody Detection in Neonatal Blood

IgG and IgM monitoring can be useful to assess past or recent infection. Some studies have assessed the dynamics of antibodies in newborns’ serum. Zeng H et al. reported that infants born to mothers with COVID-19 tested negative but had anti-SARS-CoV-2 IgM and IgG concentrations higher than normal levels at birth [57]. It is common to find maternal IgG in a newborn’s serum because IgG can pass through the placenta, providing passive immunity to the fetus. However, this is not the case for IgM, which are too large to cross the placental barrier [58]. Additionally, antibodies require at least 7 days to develop, so SARS-Co-V2 infection could have occurred early enough during pregnancy for the antibodies to be detected at birth. Although this information gives us a clue for vertical transmission, it is easily refuted because the spike protein (S), 12.5 nm in length, can cross the placenta, which allows the passage of particles of up to 250 nm length. Thus, free S proteins in the blood can pass the placental barrier and therefore induce an immune response. In the Knight et al. study, three neonates born to mothers with SARS-CoV-2 also revealed elevated levels of IgM in fetal umbilical cord blood while testing negative [17]. Given that cord blood is not representative of neonates’ blood as has been reported [59], IgM detection might originate from the mother. Alzamora et al. reported a case of a SARS-CoV-2 mother with a severe respiratory condition undergoing cesarean delivery. Sixteen hours and 48 hours post-delivery, the neonatal nasopharyngeal swab was tested positive for SARS-CoV-2 but a serological assay yielded a negative result for IgM and IgG right after birth which remained negative until the last serological test six days later [26]. These reports suggest that more studies for COVID-19 vertical transmissions need to be conducted and in this context, animal models would be very useful to univocally demonstrate whether SARS-CoV-2 can be transmitted to the fetus at birth.

## 5. Potential Mode of Action of SARS-CoV-2 Trophoblastic Evasion 

The trophoblastic evasion modes used by the virus can be predicted by extrapolating the known hijacking modes of action of SARS-CoV-2 based on trophoblastic transcriptomics. The main mechanism of viral entry is through angiotensin-converting enzyme (ACE2) /transmembrane protease, serine 2 (TMPRSS2) pathway [60], and both proteins have been observed to be expressed at the maternofetal interface. Recent data has specifically shown that they are coexpressed in the syncytiotrophoblast (SCT), villous (VCT) and extravillous (ECT) cytotrophoblast. Besides, SARS-CoV-2 was discovered to invade cells via a novel route, using the CD147/spike protein pathway [61]. Interestingly, immunohistochemistry has revealed that CD147 is expressed in the SCT, the trophoblast cells of the chorion, and in amniotic epithelial cells [62]. Thus, CD147 could also play a role in placental infection, thereby promoting fetal infection.

**Figure 1:**
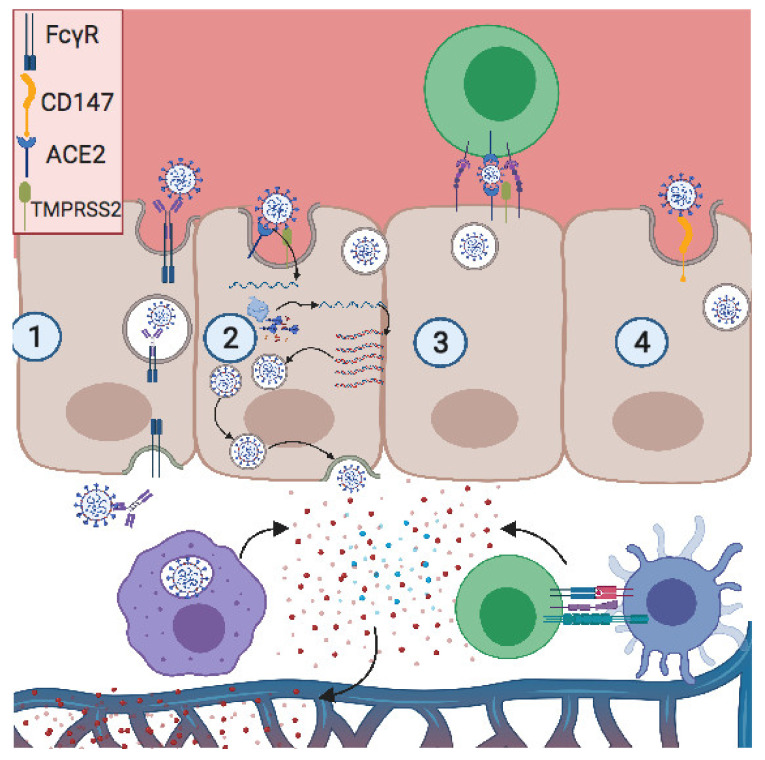
**Potential modes of placental invasion by SARS-CoV-2.** (**1**) Antibody-dependent enhancement (ADE): the antibody neutralizing SARS-CoV-2 binds its Fc region onto FCγR, expressed on the apical pole of the trophoblast. Then the neutralized virus is transcytosed into the basic pole, releasing the virus into the fetal extracellular matrix. (**2**) ACE2/TMPRSS2 pathway: the S2 subunit of the S protein interacts with ACE2 on the apical pole, promoting the fusion of the envelope with the membrane. This is followed by a proteolytic cleavage between S1 and S2 subunits by TMPRSS2, thus releasing the virus. The positive RNA translates the viral-RNA-dependent RNA polymerase and the viral proteins. Then this polymerase produces a high copy number of the viral genome, after which the virion is assembled and released on the basal pole. (**3**) Cell-to-cell contact: LFA1 expressed on infected T cells interacts with ICAM1 expressed on the trophoblast apical pole, forming a tight interaction and close proximity between two membranes. Thus, the viral release in the placental immune synapse can facilitate viral infection. (**4**) CD147 expressed on the apical pole of the trophoblast interacts with viral particles, promoting viral infection. Created with BioRender.com.

Some viruses, such as human immunodeficiency virus (HIV) and human T cell leukemia virus (HTLV), infect and spread through cell-to-cell contact. It has been demonstrated that lymphocyte function-associated antigen 1 (LFA-1) on T cells interacts with intercellular adhesion molecule (ICAM-1) on trophoblastic cells, providing cell contact and enhancing HIV transmission from maternal blood to fetal placenta [63]. SARS-CoV-2 infects T lymphocytes through spike-protein-mediated fusion [64]. As noted, trophoblastic cells express ACE2, so similarly to HIV, SARS-CoV-2 can be transferred to trophoblast via adhesion molecules, thus promoting vertical transmission through cell-to-cell contact (Figure 1).

## 6. Possible Neurological Teratogenic Effects in the Fetus by Direct SARS-CoV-2 Infection or by the Exposure to the Virus and/or Inflammation

Whether it is congenital or not, an infection raises a concern in pregnant women, because it can infect the fetus and increases the risk of teratogenicity. Currently, there is no solid evidence of vertical transmission of SARS-CoV-2. Reports that have demonstrated in utero infection, among which one demonstrated placental viremia by RT-PCR, in addition to the presence of inflammatory cells in cerebrospinal fluid (CSF), together with neurological manifestations consistent with those described in adult patients, raise concerns [23,65]. Thus, we cannot exclude the possibility of a direct viral infection of the central nervous system (CNS) in fetuses. 

### 6.1. Possible Viral Infection of the Central Nervous System 

The CNS is protected against infections thanks to many factors including the several layers of meningeal tissue surrounding the brain, the blood–brain barrier (BBB), and immunosurveillance through the resident immune cells (microglia) and patrolling of memory T cells, which enter through the choroid plexus and migrate within the CSF to then reach the lymph [66]. However, pathogens have developed evasion mechanisms to bypass these tight restrictions. 

**Figure 2:**
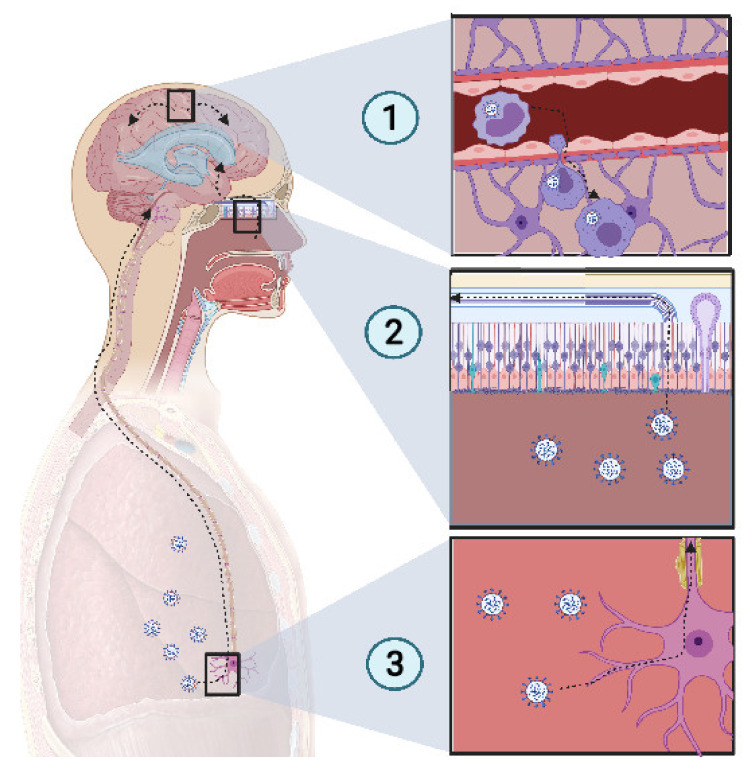
**Potential mechanism of CNS invasion by SARS-CoV-2.** (**1**) The blood−brain barrier (BBB) permits a limited exchange, due to the continuous capillary endothelium, with a tight junction supported by continuous basement membrane and surrounded by astrocyte feet. Monocytes infected with SARS-CoV-2 act as Trojan horses, due to their access to the CNS. Thus, an extravasation of infected monocytes can promote CNS infection. (**2**) The high-spectrum olfactory receptors expressed on the cilia of olfactory neurons may act as receptors for SARS-CoV-2, thus infecting the bipolar neuron in the nasal epithelium. Retrograde movement leads SARS-CoV-2 to the olfactory bulb, which is connected to the limbic system, thus promoting the invasion of the limbic system, including the hippocampus. (**3**) Chemoreceptive neuron infection in the lower respiratory tract may lead to a retrograde migration of the virus through the nervous network toward the respiratory center in the brain stem, resulting in acute respiratory failure. Created with BioRender.com

SARS-CoV-2 presents a neurotropism [67,68] as viral particles have been detected in the brains of deceased COVID-19 patients who developed neurological complications. In a study of 214 patients, Mao et al. found that CNS and peripheral nervous system (PNS) complications occurred in many COVID-19 patients. CNS complications included dizziness, headache, ischemic stroke, and intracranial hemorrhage (Figure 2).

#### 6.1.1. Pathogens Carried by Leukocytes across the BBB

Some bacterial infections, such as *Listeria monocytogenes* and *Burkholderia pseudomallei* use CD11^+^ monocytes in blood as Trojan horses because of their ability to access the CNS [66]. SARS-CoV-2 uses CD147, to mediate its infection [61,69]. It has been reported that SARS-CoV-2 infects tissue-resident CD169^+^ macrophages [70] which express CD147 receptors at their surface, the alternative pathway for SARS-CoV-2 entry. Thus, it is probable that SARS-CoV-2 could similarly invade the CNS by infecting monocytes, enabling entry to the tissue.

#### 6.1.2. Infection through Olfactory Routes

Cilia projecting from olfactory epithelium in the nasal cavity, express a wide variety of receptors that bind a large spectrum of ligands [71]. The olfactory nerve expresses a wide range of receptors, thus there is high probability of finding a matching receptor for the virus to invade the CNS through this route. When the virus accesses the olfactory nerve, it enters the olfactory bulb. Several studies have reported a loss of olfactory sense in COVID-19 patients [72,73]. Because SARS-CoV-2 is present in the nasal environment and has been shown to cause loss of olfaction, it is thought that the virus moves retrogradely from the olfactory bulb to the CNS and then spread in the brain. The olfactory bulb is part of the limbic system, which connects several brain sections, including the hippocampus. One COVID-19 patient showed a hyperintensity in the right mesial temporal lobe and hippocampus with slight hippocampal atrophy [74]. Another recent finding showed that SARS-CoV-2 was detected in the olfactory neurons of some individuals who died from COVID-19, suggesting that the virus could invade the CNS through the olfactory nerve [75].

#### 6.1.3. Retrograde Neuroinvasiveness

Several viruses have been reported to invade the CNS through retrograde movement following an invasion of the CNS. For example, replication of the rabies virus after a canine bite is followed by binding to nicotinic receptors on the motor neuron and moves centripetally without replication until it reaches the spinal cord, where it begins to replicate and to spread rapidly to the brain through retrograde movement [76]. SARS-CoV-2 has been reported to infect the respiratory center, where it can be found in high concentration [77]. Previous coronaviruses have been reported to spread via synapse from chemoreceptors and mechanoreceptors in the lower respiratory tract, reaching the cardiorespiratory center [77,78]. This suggests that the dysfunction of the respiratory center due to potential SARS-CoV-2 damage may play a role in acute respiratory failure in COVID-19 patients. If we link all of these points together, we can suppose that SARS-CoV-2, which inoculates the lower respiratory tract, could infect chemoreceptors and mechanoreceptors in the lung and pass by retrograde movement to the respiratory center in the brainstem.

#### 6.1.4. Case Report of Fetal Neuroinvasiveness of SARS-CoV-2

A study made at the University of Paris Saclay, confirmed for the first time transplacental transmission from the mother to a child in the third trimester. A SARS-CoV-2 infected pregnant woman was tested positive in the blood by RT-PCR. She underwent caesarian delivery. Amniotic fluid was collected and tested for SARS-CoV-2 RNA and tests returned positive. In order to confirm vertical transmission, nasopharyngeal and anal swabs were performed in the baby, one hour after delivery and were repeated at day three and day 18, and were positive. Bronchoalveolar lavage was collected in addition to blood sampling for RT-PCR testing and the tests also returned positive. The baby was in a bad condition and presented axial hypertonia and opisthotonos. Magnetic resonance imaging at day 11 showed bilateral gliosis of the deep white periventricular matter. After RNAemia, neurological symptoms translated into increased levels of inflammatory cells in the CSF associated with white matter injury [65]. However, this was among the rare cases of vertical transmission reporting with fetal neuroinvasiveness.

### 6.2. Cytokine Storm and Inflammation in Neonates? 

**Figure 3:**
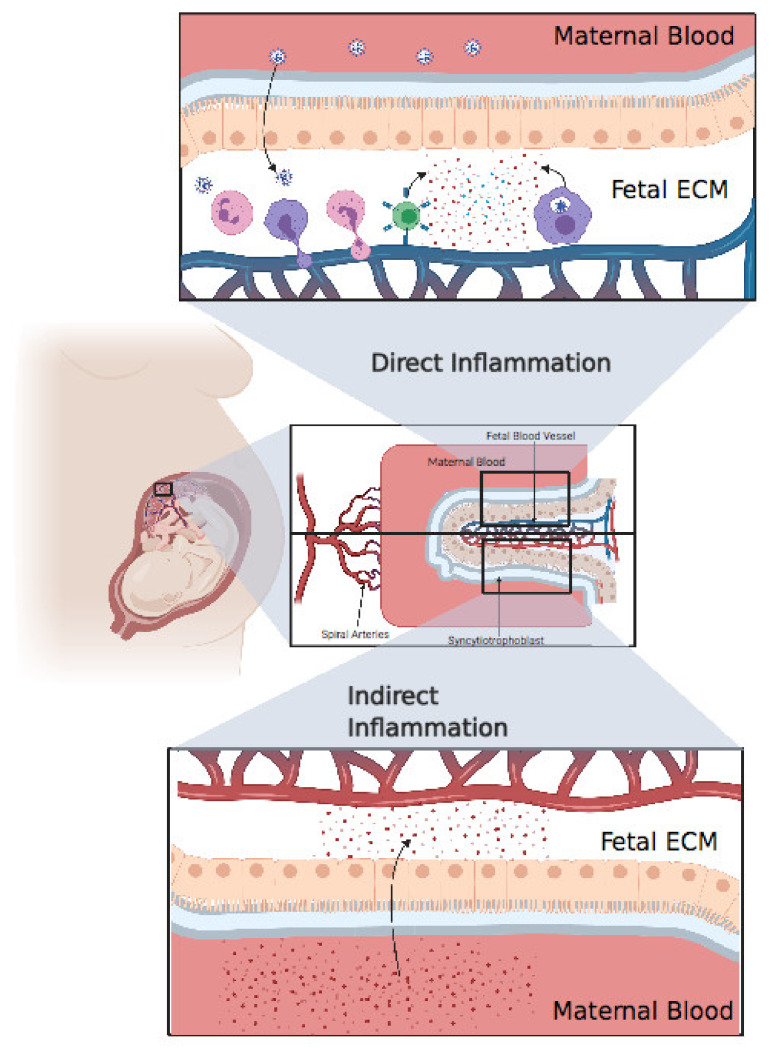
**Direct inflammation or indirect inflammation.** Direct inflammation: after viral invasion of the placenta, fetal immune cells, including neutrophils, monocytes, and T cells, extravasate from fetal blood to the placental extracellular matter, resulting in an extensive inflammatory response in the fetal extracellular matrix (ECM). Finally, these inflammatory cytokines pass through the blood, resulting in systemic inflammation in the fetus. Indirect inflammation: inflammatory cytokines circulating in maternal blood after a cytokine storm might pass through the placenta, resulting in indirect inflammation. Created with BioRender.com.

Cytokine storm is an abnormal and exaggerated immune response that results in excessive inflammation, found in graft-versus-host disease, chimeric antigen receptor T cell, autoimmune diseases, and severe viral infections [79,80]. SARS-CoV-2 certainly induces an immunopathological response but, above all, causes complications even more severe than the viral infection itself. One major complication of a cytokine storm is acute respiratory distress syndrome (ARDS), which is the main cause of death in COVID-19 patients [81]. ARDS is caused by the release of large amount of proinflammatory cytokines by immune and nonimmune cells through the activation of the Nuclear Factor Kappa B (NFκB) pathway. A pathway of NFκB activation in the coronavirus family is through activation of the pattern recognition receptor (PRR) which activates myeloid differentiation primary response 88 (MyD88) which in turn activates NFκB resulting in the production of a cocktail of proinflammatory cytokines including tumor necrosis α (TNFα) and IL-6 [82]. Virus mediated ACE2 downregulation causes dysregulation in the angiotensin II/angiotensin 1 receptor (AT1R) axis and ACE2/Mas receptor (MasR) axis resulting in the activation of complement subunits C3a and C5a resulting in decreased differentiation of T regulatory cells (Treg) and increased differentiation of T helper 17 (Th17), ultimately resulting in uncontrolled inflammatory response [83]. A 2.9-fold increase in IL-6 concentration has been found in COVID-19 patients, compared with patients with no complications [84], and IL-6 has been seen to be higher in nonsurvivors [85] (Figure 3).

These cytokines can come from different sources, namely CD4^+^ T cells and/or CD14^+^ CD16^+^ Monocytes. The cross-talk between innate immune cells and adaptive cells is maintained through these cytokines. Increased expressions of IL-6 and granulocyte monocyte colony stimulating factor (GM-CSF) was detected in COVID-19 patients [86]. Other cytokine sources during COVID-19 infection are through pyroptosis (IL6, IL1α, IL1β) [15], hemophagocytic lymphohistiocytosis (IFNγ, IL2) [87,88,89], and angiotensin II viral mimicking (IL6 positive feedback loop) [82].

Microglia is well known to contribute in neural plasticity, where it acts on sculpturing the neural network and refining neural circuitry, and a dysfunction in microglia may cause microglial neuroplasticity perturbation [90]. Microglia expressing ACE2 [91] raises thus a concern as SARS-CoV-2 can spread through this axis, leading to neuroplasticity dysfunction or even direct neuroinflammation. 

Only a few studies have assessed cytokine production in pregnant women and their neonates. A study showed that pregnant women with SARS-CoV-2 have higher inflammatory cytokine IL-6 levels compared to nonpregnant women [92]. Placental inflammation can cause fetal mortality via the release of inflammatory cytokines into fetal blood, resulting in fetal organ failure [93]. Baud et al. reported the miscarriage of a fetus in the second trimester from a mother with COVID-19, for which the placental histology revealed subchorionitis infiltrated by neutrophils and monocytes [55]. Chen Y et al. reported increased levels of IL-6, two hours after birth in all infants born from mothers infected with SARS-CoV-2. Two of these infants had elevated levels of IgM, but the PCR was negative [29]. Dong et al. also reported that a COVID-19 positive mother gave birth to a child with increased IL-6 and IgM above the baseline and a negative PCR [23]. Based on these results, we cannot conclude on the presence of fetal infection with SARS-CoV-2 [94]. The source of inflammatory cytokines may be direct, if the child is confirmed to be positive for SARS-CoV-2, or indirect through the transcytosis from maternal blood to fetal blood [95]. In either case, we must monitor the child’s health because both maternal and fetal inflammation can represent an important environmental risk factor for neurodevelopmental disorders such as schizophrenia, autistic spectrum disorder (ASD), and attention-deficit/hyperactivity disorder (ADHD) [96]. As mentioned above, it would be very important to consider developing these studies in vivo using appropriate animal models to demonstrate whether SARS-CoV-2 presents a possible threat to the emergence of neurodevelopmental disorders in children exposed to COVID-19 infection. 

### 6.3. Correlation with Neonate Abnormal Brain Development

Neonates who tested positive for SARS-CoV-2 must be monitored because the virus could have infected their CNS and caused brain damage or disrupted brain circuits, resulting in cognitive challenges or other neurodevelopmental disorders.

From another perspective, the cytokine storm discussed above and reported in neonates could also affect brain development, more specifically in preterm babies [97].

Maternal immune activation (MIA) appears to act as a neurodevelopmental disease increasing the risk of neuronal epigenetic modification resulting in neuronal disorders later in life [98]. A recent study found that maternal IL-6 was linked with decreased cognition at 12 months old, reinforcing the idea of correlation between maternal inflammation and offspring neuropsychologic disorder [99]. Hagberg et al. suggested that, not only does inflammatory reaction in the CNS have the potential to cause acute brain injury, but it also might affect brain development, causing long-term neurological consequences, hypothetically translated by disorders including cognitive impairment, schizophrenia, ASD, multiple sclerosis, cerebral palsy, and Parkinson’s disease [100]. It has been noted that during in utero infection, Lipopolysaccharide (LPS) bacteria produce pre- and postnatal brain inflammation, affecting glial cytoarchitecture and amygdala development [101]. The Danish newborn screening biobank revealed that a cytokine storm in neonates could increase the risk for ASD [102]. Moreover, neonatal encephalopathy is associated with increased levels of IL-6, IL-8, and IL-1β [103]. Furthermore, recent studies have shown that placental infection was associated with high levels of IL-6 and IL-8 during parturition, and elevated IL-6, IL-8, IL-1β, and TNFα in the umbilical cord could worsen neurological outcomes after six months [104]. Perinatal inflammation during the developmental phase can affect the brain during the fetal period as well as over a long period in postnatal life, where it can affect cortical plasticity and myelination, thus producing an adverse effect on neural connections and the rate of neural message delivery [105]. Phosphoinositide 3-kinase δ (PI3Kδ) inhibitors which are used as a therapeutic target for the inhibition of proinflammatory cytokines can be used to suppress a cytokine storm, and consequently might stop the drastic negative effect that inflammation can cause on neonatal brain development [106].

## 7. Limitations of the Current Studies 

In this review, we provided an objective analysis regarding vertical transmission of SARS-CoV-2, but none of the articles found presented conclusive results. For example, even the infants who tested positive using RT-PCR [19], were tested 36 h after delivery; thus they had the potential to be infected postnatally. Positive RT-PCR tests for the three neonates diagnosed by Zeng L et al. were performed on days 2, 4, 6, and 7 after birth. 

The recent review that analyzed 84 retrospectives studies to seek proof of vertical transmission cited previously has taken into consideration new parameters for their analyses like diagnostic analyses, placental analyses and the scientific limitations in interpreting data and not just RT-PCR results in babies. Diagnostic testing combined with incubation period and placental pathology indicates a strong likelihood of a vertical transmission of SARS-CoV-2 from mother to baby is possible [34]. However, we still lack confirmation of vertical transmission and this study underlines the importance of the combination of several parameters in our studies and sometimes it is very complicated to have access to all the information, hence the necessity to develop a new accessible model such as an animal model. 

Aside of vertical transmission, the question that must be asked is why neonates born from infected pregnant women but testing negative for SARS-CoV-2 have experienced fetal distress [19,20,107] and multiple organ failure [12,13,95,107]? It is therefore possible that the fetus by exposure to the maternal inflammatory environment, might have developed these complications. Thus, the primary concern in pregnant women might not be the virus itself but the cytokine storm that could directly impact the fetus and cause these detrimental effects. 

To date, reports that confirm vertical transmission are very scare and nonconclusive. From the literature, we were not able to compile enough data and convincing studies reporting SARS-CoV-2 vertical transmission. However, we believe that fetal exposure to an inflammatory environment might have fatal consequences on child neurodevelopment, by causing ASD or ADHD. Therefore, fetal generated complications might be due to the consequences from the mother’s viral infection. 

Neonates from mothers infected with the novel coronavirus or children exposed to SARS-CoV-2 must be subjected to long-term monitoring. More studies are needed and early intervention to assess whether a maternal cytokine storm affects fetal brain development must be implemented. 

## 8. How Can Animal Models Help Us to Resolve the Question of the Vertical Transmission, and the Impact of Inflammation on the Brain during Neurodevelopment?

Longitudinal studies on both mothers and children several months after birth are required in order to evaluate the indirect impact of SARS-CoV-2 on the developing fetus. Such studies will determine whether some of the fetal complications discussed above, and particularly brain disorders, could be due to an indirect inflammation caused by the transport of inflammatory cytokines from the mother to the fetus.

As soon as the COVID-19 epidemic started, worldwide efforts from experts in the field were made to come up with some relevant experimental models [108]. Animal models that recapitulate the hallmark features of the human disease are valuable in elucidating pathogenic mechanisms, identifying new therapeutic targets, developing and testing new and effective treatments. Importantly, researchers are now able to integrate physiology, behavior, neuroimaging, and postmortem studies to explore the mechanisms of neurodevelopmental disorders.

The intense research into SARS-CoV-2 since the epidemic onset has come up with different models including mice, hamsters, ferrets, and nonhuman primate (NHP) models. Briefly, gold standard mice are not sensitive to natural infection with the initial virus due to the lack of appropriate ACE2 receptors that bind to the viral spike protein [109]. Strategies have been developed and mouse models of mild and severe COVID-19 are now available thanks to virus adaptation to mouse ACE2 or genetically modified mice expressing human ACE2 [107,110,111,112]. Recently, natural variants of the virus have been shown to efficiently infect mice with high replicating titers in lungs, resulting from acquired changes in the spike protein to adapt to the mouse ACE2 [113]. Ferrets present undetectable or mild symptoms of a predominantly upper respiratory tract with efficient contact transmission of the disease, but there is a lack of tools for the species in order to comfortably explore this model [114,115,116]. Similar observations apply to the hamster model with mild to moderate disease, age- and sex-related severity as seen in human population and efficient transmission between individuals [117,118,119,120]. NHPs and especially macaques species (*M. fascicularis* and *M. mulatta*) are susceptible to SARS-CoV-2 infection and display high levels of viral replication for seven days in both the upper and lower respiratory tracts, with viral shedding in respiratory and gastrointestinal tracts exacerbated by age [121,122,123]. NHPs present pathological features of viral pneumonia and a variable induction of either mild clinical disease symptoms in cynomolgus macaques [124] while rhesus macaques develop moderate symptoms [125], and a robust protection against rechallenge has been reported in this model [126]. SARS-CoV-2 infection models in macaques are therefore useful for evaluating the efficacy of vaccines and effective treatments [127,128,129,130,131].

Yet to date, no in vivo infection model during gestation, has been reported. An animal model in this context would be valuable as it would allow the evaluation of the risk of vertical transmission of the SARS-CoV-2 infection and the impact of the maternal cytokine storm on the neonatal and postnatal development of the fetus, and more specifically on its neurodevelopment. NHPs may be particularly relevant to evaluating immune-based prenatal risk factors given the similarities with humans in gestational and neurodevelopmental timeline, immune systems, uterine anatomy, singleton gestation, hemochorial placentation, hormonal control of parturition and microbial communities within the vagina [26]. In this regard, it is noteworthy that innovative centers dedicated to infectious disease models and therapies such as the IDMIT (CEA, Fontenay-aux-Roses) are valuable. An international Pediatric Immunology Program network has recently been set up to develop and explore pediatric immunity in infectious diseases using the NHP models. Intrauterine infection, for example, can be achieved in the NHP model utilizing chronically catheterized monkeys with timed gestations by intra-amniotic inoculation, then allowing longitudinal samples of maternal and fetal blood and amniotic fluid, fetal heart rate and maternal temperature along with uterine activity monitoring with indices of infection and inflammatory mediators [132,133]. Recent studies from IDMIT have successfully established two neonate-NHP models for in utero Zika virus and postnatal SARS-CoV-2 infections respectively (manuscripts in preparation). Furthermore, NHP models have been developed for in utero bacterial and viral infectious diseases or inflammatory-state for physiopathological studies purposes or treatment strategies evaluation [134,135,136,137,138].

Studies on the impact of a SARS-CoV-2-induced cytokine storm on fetal neurodevelopment are challenging, as there is no data on the threshold value for cytokine concentrations nor a timeframe to induce brain developmental disorders. These disorders are characterized by deficits in a range of complex cognitive, social, and affective functions in the human brain areas, such as the prefrontal cortex, which involves high cognitive functions that need to be explored in an appropriate animal species [139]. Primates exhibit an expansion of the prefrontal cortex during evolution, which is considered one of the key regions for regulating social cognition [140]. By combining some keys factors such as (i) similarities between animal and human stages of brain development; (ii) quantifiable biomarkers of postnatal functional alterations (motor or evolutionary cognitive deficits); (iii) correlations between in vivo imaging and in vitro data outcomes, we may have the ability to detect and monitor some neurodevelopmental alterations and further evaluate the therapeutic strategies that are needed to correct or compensate for these alterations.

Primates share matching stages of brain maturation with gestational age due to a long brain maturation process that takes place during gestation and long after birth [141,142]. Moreover, macaques live in a complex, hierarchical social system and use many forms of human-like communication means such as facial expressions and social gestures. This rich social and cognitive repertoire provides a framework to relate behavioral changes observed in the animal model more directly to human mental illness compared to rodent species [139], with neonate cognitive and motor development being well characterized through transposed human neonatal tests [143].

SARS-CoV-2 experimental models during gestation will have to explore whether the mild and moderate forms of COVID-19 observed in NHP models, induce an inflammation with enough cytokines to impact brain development. Modelling of severe forms could be required, mainly relying on comorbidity factors such as hypertension, diabetes, age and obesity. MIA models could also be essential tools for exploring the relationship between immune activation and neural development, regardless of the infectious agent. In the mouse models it has been reported that in the absence of an infectious agent, immune stimulations induced by some bacterial or viral products or soluble cytokines were sufficient to trigger an active immune response in the pregnant dam, leading to some anxiety, impaired social and repetitive behaviors in the offspring [144,145]. In the NHP model, Short SJ et al. reported that rhesus offspring born to mothers exposed to influenza in the early third trimester show reduced gray matter volume throughout the cortex and increased white matter in the parietal cortex at 1 year of age with no direct viral exposure [146]. In Bauman’s lab, a 4-years longitudinal study of a rhesus MIA model indicates brain alterations and behavioral development of offspring, increased striatal dopamine in late adolescence and persistent immune dysfunction as the animals aged [139].

Whereas SARS-CoV-2 induces an inflammatory state during pregnancy with specifically increased levels of IL-6, studies already reported that a prolonged exposure to elevated amniotic fluid IL-6 induce a subacute or a chronic inflammation in fetal lungs with neonatal sequels such as bronchopulmonary dysplasia [138]. Mouse models also demonstrated that placental IL-6 signalling is required for MIA-induced acute immune activation in the fetal brain, as well as downstream neuropathologies and behavioral impairments. These data suggest a predominant role of the interactions at the materno-placental-fetal interface in relaying the effects of maternal gestational insults to the developing embryo and underlie potential preventive therapeutic approaches in immune-induced brain developmental disorders [147]. 

## 9. Conclusions

After a large analysis of the literature searching for evidence that SARS-CoV-2 infection leads to direct vertical transmission, there is a possibility but we still lack evidence and the question remains open. Even if children are less susceptible to disease complications with mainly mild symptoms, pediatricians are not fully aware of the possible long-term effects of inflammation and/or preterm delivery on brain development. In this review, we suggested that MIA or a cytokine storm might have an impact on fetal brain development. Thus, close monitoring and early intervention in young children born to infected mothers would be highly recommended, especially in the case of preterm babies. To address these questions, one possibility could be the use of preclinical models. We thus urgently need to develop some animal models to further explore vertical transmission and the possibility of neurodevelopmental disorders. These models will allow us to make the link between SARS-COV-2 infection, inflammation and the impact on brain development of the fetus with a long-term follow-up. Another possibility is to prioritize the vaccination of women planning for pregnancy. This subject is very important and can be easily addressed by using a preclinical model. 

## Data Availability

It is a review of literature, there is no experimental data done by the authors.
